# Trends and variations in the prescribing of secondary preventative cardiovascular therapies for non-ST elevation myocardial infarction (NSTEMI) in Malaysia

**DOI:** 10.1007/s00228-018-2451-3

**Published:** 2018-03-26

**Authors:** Padmaa Venkatason, Nur Lisa Zaharan, Muhammad Dzafir Ismail, Wan Azman Wan Ahmad, Ahmad Syadi Mahmood Zuhdi

**Affiliations:** 1 Department of Medicine, Faculty of Medicine, University of Malaya, 50603 Kuala Lumpur, Malaysia; 2 Department of Pharmacology, Faculty of Medicine, University of Malaya, 50603 Kuala Lumpur, Malaysia

**Keywords:** Prescribing, Preventative cardiovascular, STEMI, NSTEMI, Inequality

## Abstract

**Purpose:**

Information is lacking on prescribing of preventative cardiovascular pharmacotherapies for patients with non-ST elevation myocardial infarction (NSTEMI) in the Asian region. This study examined the prescribing rate of these pharmacotherapies, comparing NSTEMI to STEMI, and variations across demographics and clinical factors within the NSTEMI group in the multi-ethnic Malaysian population.

**Methods:**

This is a retrospective analysis of the Malaysian National Cardiovascular Disease Database-Acute Coronary Syndrome registry from year 2006 to 2013 (*n* = 30,873). On-discharge pharmacotherapies examined were aspirin, ADP-antagonists, statins, ACE-inhibitors, angiotensin-II-receptor blockers, and beta-blockers. Multivariate logistic regression was used to calculate adjusted odds ratio of receiving individual pharmacotherapies according to patients’ characteristics in NSTEMI patients (*n* = 11,390).

**Results:**

Prescribing rates for cardiovascular pharmacotherapies had significantly increased especially for ADP-antagonists (76%) in NSTEMI patients. More than 85% were prescribed statins and antiplatelets but rates remained significantly lower compared to STEMI. Women and those over 65 years old were less likely to be prescribed these pharmacotherapies compared to men and younger NSTEMI patients. Chinese and Indians were more likely to receive selected pharmacotherapies compared to Malays (main ethnicity). Geographical variations were observed; East Malaysian (Malaysian Borneo) patients were less likely to receive these compared to Western region of Malaysian Peninsular. Underprescribing in patients with risk factors such as diabetes were observed with other co-morbidities influencing prescribing selectively.

**Conclusion:**

This study uncovers demographic and clinical variations in cardiovascular pharmacotherapies prescribing for NSTEMI. Concerted efforts by policy makers, specialty societies, and physicians are required focusing on elderly, women, Malays, East Malaysians, and high-risk patients.

**Electronic supplementary material:**

The online version of this article (10.1007/s00228-018-2451-3) contains supplementary material, which is available to authorized users.

## Introduction

Antiplatelets, beta-adrenoceptor blockers (beta-blockers), ACE inhibitors (ACEIs) or angiotensin-II-receptor blockers (ARBs), and statins are recommended as secondary preventative cardiovascular (CV) pharmacotherapies in post- acute myocardial infarction (AMI) patients by international clinical practice guidelines [1, 2]. Although similar therapies were indicated for both ST-elevation MI (STEMI) and non-STEMI (NSTEMI), under-prescribing in prescribing for NSTEMI patients were shown in Europe and USA [3–7] highlighting the need to examine prescribing trends in other parts of the world. Previous studies have also demonstrated variations in CV prescribing especially in women, the elderly and ethnic minorities and across geographical regions [8, 9]. However, clinical factors remained as primary determinants of prescribing. Thus, variations in prescribing for NSTEMI across different demographics needs to be explored alongside clinical factors to identify possible sources of inequalities for improvement of care.

Malaysia, a multi-ethnic, upper middle-income country in South-East Asia, provides a unique opportunity to study prescribing trends in this region of the world. Geographically, Malaysia is made up of two parts separated by the South China Sea; the Malaysian Peninsular (West Malaysia) and the East of Malaysia on the northern part of Borneo (Malaysian Borneo) [10]. The three main ethnicities are Malays (50%) followed by Chinese (23%) and Indians (7%) while the rest are indigenous natives and other non-citizens [11]. The ethnicities differ in terms of culture, religion and socioeconomic statuses where the Chinese had economic advantages while the Malays hold political power [12]. Malaysia has expanded taxation-based public healthcare delivery system to provide access to comprehensive and affordable services via government-run healthcare facilities [13, 14]. Parallel to this, there are fast-growing private hospitals and clinics that are mostly concentrated in urban areas [14]. There are also corporatized public entities, one example being the National Heart Institute, at the crossroad of both public and private sectors [15].

Despite embracing advances in management of cardiovascular (CV) disease, it remained the leading cause of mortality in Malaysia, accounting for 23% of all deaths [16]. However, little information is available on the prevalence of STEMI and NSTEMI [17]. An increase in the prescribing of evidence-based pharmacotherapies has been described for STEMI [18] while less is known on NSTEMI. In acute interventions of AMI, women [19] and elderly patients [20] were less likely to receive optimal management compared to men and younger patients while no significant variations across ethnicities were observed [21]. Regional variations have remained unexplored. Exploring regional variations alongside other demographic variations will allow identification of gaps in provision of equal services across the country [22].

This study was aimed to examine trends in the prescribing of on-discharge secondary preventative CV therapies for patients with AMI, comparing STEMI and NSTEMI in Malaysian population using the prospective National Cardiovascular Disease Database-Acute Coronary Syndrome (NCVD-ACS) registry. This study further focused on variations in prescribing for NSTEMI patients across age groups, gender, geographical regions, clinical risk factors and co-morbidities. This registry is part of the NCVD registry whereby 18 hospitals contributed data from patients with CV diseases from year 2006 till current. It is being sponsored by the National Heart Association of Malaysia (NHAM) and are continuously monitored to ensure quality. The registry included information on demographic information, CV diagnosis, co-morbidities, family history and in-hospital management and on-discharge medications. The details of the registry have been described in detail elsewhere [23]. This database is among the few in South East Asia and thus provided valuable information on CV disease in this region of the world.

## Methodology

### Study population

Patients with AMI (*n* = 30,873), both STEMI (*n* = 19,483) and NSTEMI (*n* = 11,390) were identified from the NCVD-ACS registry from year 2006 to year 2013. Variables extracted were gender, age, ethnicity, calendar year of visit, risk factors such as diabetes mellitus, hypertension, smoking status, family history of ischemic heart disease (IHD), previous history of IHD, and co-morbid conditions such as cerebrovascular disease, peripheral vascular disease (PVD), chronic kidney disease (CKD) and chronic lung disease. On-discharge CV medications included were antiplatelets (aspirin and adenosine diphosphate (ADP)-antagonists), ACEIs or ARBs, beta-blockers, and statins.

### Statistical analyses

Categorical variables were presented as frequency and percentages. Univariate analysis was used to compare characteristics between STEMI and NSTEMI patients. Prescribing rate of each secondary preventative CV pharmacotherapies were calculated according to calendar years and linear test was used to examine changes over time. Prescribing variations across groups of interest in NSTEMI patients were examined using binary logistic regression adjusting for covariates and presented as odds ratio (OR) and 95% confidence intervals (CI). Covariates chosen were those shown to impact prescribing trend such as age group, gender [19], ethnicity [9], calendar year, geographical regions [22], presence of risk factors and presence of co-morbidities [1, 2]. For geographical region, the Western Peninsular was chosen as the reference as this region has the most tertiary centers for CV referrals in this country [24]. SPSS version 24 was used for statistical analyses (IBM SPSS Statistics, NY). *P* < 0.05 was considered statistically significant.

## Results

**Fig. 1:**
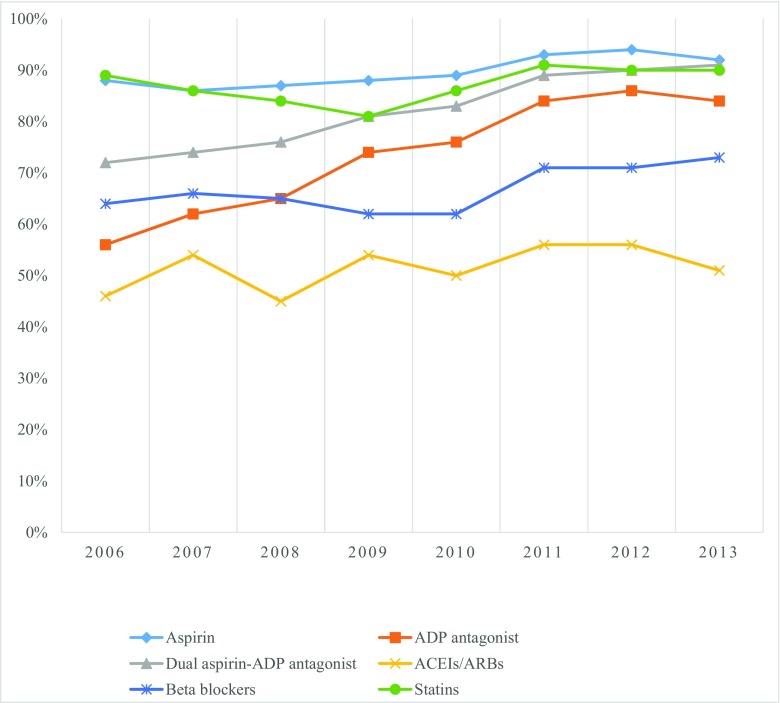
Trends in the prescribing of on-discharge secondary preventative cardiovascular pharmacotherapies in patients with AMI in the Malaysian NCVD-ACS registry from 2006 to 2013

There were 30,873 patients who presented with AMI during this study period (81% males, 70% less than 65 years old and 53% Malays) (Table 1). Of those, 37% were NSTEMI. There were significant differences in demographics, CV risk factors and co-morbidities, comparing STEMI and NSTEMI (Table 1). Significantly lower proportion of NSTEMI patients received antiplatelets (*p* < 0.0001), beta-blockers (*p* = 0.001), and statins (*p* = 0.002) compared to STEMI (Supplementary Table 1). Significant increases in the prescribing of these agents (*p* < 0.0001) were observed over the study period (Fig. 1) with marked increase in the prescribing of ADP-antagonists. The prescribing of dual aspirin-ADP antagonists increased in parallel.

**Table 1:** Baseline characteristics of patients who presented with AMI in the Malaysian NCVD-ACS between 2006 to 2013, comparing STEMI and STEMI

Characteristics	Total *n*, (%)	STEMI *n*, (%)	NSTEMI *n* (%)	*p* value
Age				
< 65	21,016 (70%)	14,463 (76%)	6553 (60%)	< 0.001
≥ 65	8976 (30%)	4509 (24%)	4467 (40%)	
Gender				
Male	24,999 (81%)	16,672 (86%)	8327 (73%)	< 0.001
Female	5874 (19%)	2811 (14%)	3063 (27%)	
Ethnicity				
Malay	16,431 (53%)	11,276 (58%)	5155 (45%)	
Chinese	6215 (20%)	3454 (18%)	2761 (24%)	< 0.001
Indians	6151 (20%)	3270 (17%)	2881 (25%)	
Others	2076 (7%)	1483 (8%)	593 (5%)	
Risk factors				
Previous IHD	4964 (19%)	2126 (13%)	2838 (30%)	< 0.001
Diabetes	12,882 (48%)	7065 (43%)	5817 (56%)	< 0.001
Hypertension	17,778 (65%)	9706 (58%)	8072 (76%)	< 0.001
Dyslipidaemia	9637 (41%)	4927 (35%)	4710 (50%)	< 0.001
Smoking	12,442 (54%)	9609 (63%)	2833 (36%)	< 0.001
Family History	3469 (11%)	2195 (11%)	1274 (11%)	< 0.001
Co-morbidities				
Cerebrovascular disease	1020 (4%)	514 (3%)	506 (5%)	< 0.001
Peripheral vascular disease	222 (1%)	55 (< 1%)	167 (2%)	< 0.001
CKD	2112 (8%)	646 (4%)	1466 (15%)	< 0.001
Chronic lung disease	880 (3%)	400 (2%)	480 (5%)	< 0.001
Regions				
Western Peninsular	11,840 (39%)	5837 (31%)	6003 (53%)	< 0.001
Eastern Peninsular	4183 (14%)	3143 (16%)	1040 (9%)	< 0.001
Northern Peninsular	7084 (23%)	4565 (24%)	2519 (22%)	< 0.001
Southern Peninsular	4184 (14%)	3625 (19%)	559 (5%)	< 0.001
East Malaysia	3104 (10%)	1931 (10%)	1173 (10%)	< 0.001

Older patients and women with NSTEMI were less likely to receive CV therapies (except beta-blockers) compared to younger patients and men (Table 2). Chinese and Indians were more likely to receive ADP-antagonists compared to Malays. Chinese were also more likely to receive statins while Indians were more likely to receive ACEIs/ARBs. There were significant regional differences, for example, patients in the Northern peninsular were more likely while those in East Malaysia were less likely to be prescribed these agents compared to Western peninsular. Patients with previous IHD or diabetes were less likely to be prescribed aspirin and statins compared to those without (Supplementary Table 2). Those with hypertension were more likely to receive ACEIs/ARBs and beta blockers. CKD patients were less likely to receive aspirin, ACEIs/ARBs and statins, those with cerebrovascular disease were less likely to be prescribed aspirin while those with chronic lung disease were less likely to receive beta-blockers.

**Table 2:** Demographic variations in the prescribing of on-discharge secondary preventative cardiovascular pharmacotherapies for patients with NSTEMI in the Malaysian NCVD-ACS registry (2006–2013) presented as adjusted odds ratio (OR) with 95% confidence interval (CI)

Demographic characteristics	Aspirin		ADP-antagonists		ACEIs/ARBs		Beta blockers		Statins	
	*N*, %	OR (95% CI) *p* value	*N*, %	OR (95% CI) *p* value	*N*, %	OR (95% CI) *p* value	*N*, %	OR (95% CI) *p* value	*N*, %	OR (95% CI) *p* value
Age										
< 65	5443 (91%)	Reference	3300 (72%)	Reference	3526 (55%)	Reference	3995 (70%)	Reference	5192 (88%)	Reference
≥ 65	3438 (85%)	0.54(0.47, 0.61) *	2180 (70%)	0.89(0.81, 0.99)*	2114 (49%)	0.79 (0.73,0.85) *	2488(63%)	0.75 (0.68, 0.81)*	3412(85%)	0.77(0.68, 0.86)*
Gender										
Male	6809 (90%)	Reference	4202 (74%)	Reference	4381 (54%)	Reference	4970 (68%)	Reference	6533 (87%)	Reference
Female	2359 (85%)	0.65(0.57, 0.74) *	1450 (66%)	0.70(0.63, 0.77) *	1461 (50%)	0.85 (0.78,0.92)*	1726 (64%)	0.84 (0.77, 0.93) ns	2351 (86%)	0.89(0.78, 1.01) ns
Ethnicity										
Malay	4148 (89%)	Reference	2484 (69%)	Reference	2647 (53%)	Reference	3063 (68%)	Reference	3991 (87%)	Reference
Chinese	2220 (88%)	0.95(0.81, 1.08) ns	1373 (73%)	1.15(1.01, 1.27) *	1392 (52%)	0.89 (0.74, 0.99) 0.002	1633 (67%)	0.94(0.84, 1.09) ns	2168 (87%)	1.12(1.01, 1.28) *
Indian	2359 (90%)	1.05(0.94, 1.12) ns	1544 (74%)	1.25 (1.10, 1.36) *	1574 (57%)	1.14(1.01, 1.25) *	1692 (66%)	0.87(0.79, 0.98) 0.007	2311 (88%)	1.20 (1.09, 1.32) *
Others	441 (89%)	0.98 (0.89, 1.15) ns	251 (70%)	1.04(0.92, 1.17) ns	229 (39%)	0.65 (0.49,0.79) *	308 (64%)	0.74(0.60, 0.87) 0.0001	414 (84%)	0.88 (0.74,0.96) 0.002
Regions										
Western	4894	Reference	3206	Reference	3032	Reference	3294	Reference	4653	Reference
Peninsular	(89%)		(70%)		(53%)		(63%)		(87%)	
Eastern	842	0.96 (0.85, 1.08)	523	0.95(0.81, 1.14)	664	1.45(1.32,1.58)	729	1.57(1.45, 1.72)	821	0.94(0.82,1.08)
Peninsular	(88%)	ns	(69%)	ns	(64%)	*	(76%)	*	(86%)	ns
Northern	2177	1.07(0.94, 1.21)	1205	1.14(1.01, 1.28)	1539	1.34 (1.23, 1.42)	1777	1.41 (1.32, 1.57)	2124	1.12(1.01, 1.38)
Peninsular	(92%)	ns	(77%)	*	(61%)	*	(75%)	*	(89%)	0.001
Southern	362	1.01(0.92, 1.15)	187	1.24(1.12, 1.36)	170	0.51 (0.38, 0.69)	240	1.10 (1.02, 1.23)	400	1.25(1.10, 1.41)
Peninsular	(91%)	ns	(81%)	*	(31%)	*	(63%)	0.004	(91%)	*
East Malaysia	856 (83%)	0.68 (0.58, 0.74) *	523 (67%)	0.82(0.70, 0.97) *	416 (36%)	0.69 (0.51, 0.78) *	637 (63%)	1.01(0.91, 1.20) ns	845 (82%)	0.81(0.74, 0.94) *

*ns* not significant

*p < 0.0001

## Discussion

There has been a significant increase in the prescribing of all CV therapies during the study period, with more than 85% of Malaysian patients with NSTEMI being prescribed aspirin and statins. The highest increase was observed with ADP-antagonists. NSTEMI patients were less likely to receive these medications compared to STEMI. Women and those ≥ 65 years old were less likely to receive CV therapies compared to men and younger NSTEMI patients. Significant variations were found across ethnicities and geographical regions. Risk factors such as diabetes and hypertension and co-morbidities such as cerebrovascular disease, CKD and chronic lung disease influenced CV prescribing for these patients.

Improvement in prescribing rate is similarly observed in other countries [7, 25, 26] and is believed to contribute to improvement in NSTEMI outcomes [27, 28]. Similar trend has been described for STEMI patients [18]. This may be due to increased adherence to clinical guidelines especially in hospitals who participated in NCVD registry. The Malaysian MOH together with NHAM are active in promoting evidence-based therapies and provided easy access to local clinical practice guidelines [29], both online and as small handbooks distributed throughout hospitals in Malaysia. Cost of medications may have influenced prescribing. Within each class of therapies are patented and generics drugs and efforts to increase generic formulations in Malaysia may improve availability of these drugs.

Like other population, women and the elderly were less likely to receive CV therapies compared to men and younger patients [8, 30]. Under-prescribing in the elderly has been described as “treatment-risk” paradox whereby patients become less likely to receive appropriate treatment with increasing age [31]. Financial consideration may play a role, especially in those who opt for non-generic drug [32]. Interestingly, Malaysians presented with MI at younger age compared to other developed countries [21]. Gender disparities may be explained by lower perceived risk of MI for women [33]. Malaysian women with MI were significantly older as well as having higher rates of co-morbidities compared to men [34]. The greatest CV treatment benefit for mortality reduction occurred in women between 65 and 84 years old [28]; hence, this group needs special attention. Reports of under-prescribing of medications in women are not specific to cardiovascular diseases and may require far-reaching measures in health care planning.

Chinese and Indians were more likely to receive CV therapies compared to Malays as the main ethnicity. Different ethnicities may exhibit different clinical profiles, for example, Chinese had highest rate of hypertension and hyperlipidemia while Indians had higher rate of diabetes [21]. Interestingly, both ethnicities have lower risk of cardiovascular mortality compared to Malays for NSTEMI [21]. Ethnic differences may reflect socioeconomic differences [9, 35]. Malays were generally concentrated in the poorer socioeconomic quintiles and hence considered to be socioeconomically disadvantaged [36]. Prescribing for other ethnic minorities was not significantly different to the main ethnicity. In contrast, Caucasians as the main race were more likely to receive medications compared to Hispanics, African Americans and Asian Americans in the USA [37]. The East Malaysia region, which is separated from the Malaysian peninsular, was less likely to receive these medications. Regional variations may be explained by characteristics of individuals and area-level factors such as population health, education levels, and ethnic composition [22] in addition to preference of hospitals and individual physicians [5]. There were a mix of ethnic minorities living in this region with lower socioeconomic status [12, 14, 36] and this may have influenced prescribing.

Those with NSTEMI were less likely to receive these medications compared to STEMI as physicians may favor more aggressive preventative therapies for STEMI [38]. Differences in demographic and clinical factors between these two groups may affect prescribing. Presence of clinical risk factors affected treatment preference for NSTEMI. For example, patients with hypertension were more likely to receive ACEIs/ARBs and beta-blockers. Surprisingly, those with previous history of IHD or diabetes were less likely to receive CV therapies compared to those without. This may be explained by ‘risk-treatment paradox’ where patients with higher risk of CVD were less likely to receive evidence based treatment [39]. Possible reason includes gaps in the evidence whereby there was uncertainty about the risk: benefit ratio in patients at higher risk. There may be information gaps whereby additional information not captured in the database may explain possible confounding factors in clinical decisions [39]. Concerns about co-morbidities such as chronic lung disease and CKD influenced prescribing options. Local specialty society such as NHAM have a role in promoting and educating physicians, especially junior physicians on current evidences and best practice [40].

This study uncovered variations in prescribing of evidence-based pharmacotherapies in NSTEMI patients using national CV registry. Recommendations for improvement should include establishment of hospital-based approaches such as medication checklists [41]. Information technology should be integrated to support decision-making and allows in-hospital prescribing to be followed up at discharge [42]. Ideally, treatment plan should be communicated with primary care physicians for continuity of care. Malaysia have not embraced the one patient-one GP system [14]. However, these patients will be followed up at each hospital’s outpatient clinic. Regular audit activities should be undertaken to ensure clinical practices are in line with guidelines and identify inequalities within hospital [43]. The state plays a role in promoting equal standard of care and ensuring continuous access to all evidence-based pharmacotherapies [14]. More generic medicine with good efficacy should be introduced as alternative to expensive originals, even in patients who attended private centers for a more efficient use of health resources [44]. Training for more physicians in the cardiology specialty to be based in different state hospitals should also be considered. In 2014, the number of cardiologists practicing in private centers in Malaysia were 184, compared to only 35 cardiologists in the government sector [24]. Physicians in general exclusively work in either state/government hospitals or private practice [14] although a trend toward dual practice is emerging [15]. This unequal distribution of physicians may influence prescribing trends in the country.

### Strength and limitations

This study utilized data from hospitals nationwide to represent unselected group of patients in a real-world setting. The inclusion of different spectrum of patients allows prescribing trends to be examined objectively. The database is well maintained, and training is provided regularly to ensure data quality. Some information is self-reported such as smoking status and past medical history and thus misclassification and under-reporting may occur. Information that measures socioeconomic status such as occupation and educational levels were not available. Ethnic minorities living in remote areas may have difficulty accessing health facilities and could have been under-represented. Participation of hospitals into this database is voluntary and thus there may be a selection bias and an overestimate of prescribing in the general Malaysian population. Many private hospitals did not participate in this registry and thus prescribing in this sector could not be determined.

## Conclusion

In conclusion, the prescribing of secondary preventative cardiovascular therapies for patients with NSTEMI had demonstrated significant improvement. This study uncovered demographical and clinical variations in the prescribing of cardiovascular therapies and hence concerted efforts by policy makers, specialty societies and physicians are required to overcome inequalities. Target population should include women, the elderly, Malay ethnicity, East Malaysians, diabetes, and CKD patients.

## Electronic supplementary material

ESM 1 (DOCX 19 kb)
