# Supplementary material for: Trends and variations in the prescribing of secondary preventative cardiovascular therapies for non-ST elevation myocardial infarction (NSTEMI) in Malaysia
Source: Eur J Clin Pharmacol. 2018 Mar 26;74(7):953–60. doi: 10.1007/s00228-018-2451-3 (PMC5999133; doi:10.1007/s00228-018-2451-3)
Supplement: Supplementary file 1 — (DOCX 19 kb) [file 228_2018_2451_MOESM1_ESM.docx]

Supplementary Table 1: Prescribing of secondary preventative cardiovascular pharmacotherapies for patients with AMI in the Malaysian NCVD-ACS registry (2006-2013), comparing STEMI and NSTEMI

| **Cardiovascular pharmacotherapies** | **Total**  **n, (%)** | **STEMI**  **n, (%)** | **NSTEMI**  **n (%)** | ***p*-value** |
| --- | --- | --- | --- | --- |
| Antiplatelets |  |  |  |  |
| - Aspirin | 24,826 (91%) | 15,658 (92%) | 9,168 (90%) | <0.0001 |
| - ADP-antagonists | 15,977 (76%) | 10,325 (78%) | 5,652 (72%) | <0.0001 |
| - Dual antiplatelets | 15,370 (71%) | 10,096 (75%) | 5,274 (65%) | <0.0001 |
| ACEIs/ARBs | 15,826 (52%) | 9,984 (52%) | 5,824 (53%) | ns |
| Beta Blockers | 18,039 (68%) | 11,343 (69%) | 6,696 (67%) | 0.001 |
| Statins | 23,927 (88%) | 15,043 (88%) | 8,884 (87%) | 0.002 |

Ns: not significant

Supplementary Table 2: Clinical variations in the prescribing of on-discharge secondary preventative cardiovascular pharmacotherapies for patients with NSTEMI in the Malaysian NCVD-ACS registry (2006-2013) presented as adjusted odds ratio (OR) with 95% confidence interval (CI)

| **Clinical characteristics** | **Aspirin** | | **ADP-antagonists** | | **ACEIs/ARBs** | | **Beta blockers** | | **Statins** | |
| --- | --- | --- | --- | --- | --- | --- | --- | --- | --- | --- |
|  | N, % | OR (95% CI)  *p-*value | N, % | OR (95% CI)  *p-*value | N, % | OR (95% CI)  *p-*value | N, % | OR (95% CI)  *p-*value | N, % | OR (95% CI)  *p-*value |
| **Risk Factors∞**  Previous IHD  Diabetes  Hypertension  Dyslipidaemia  Smoking  Family History | 2292 (87%)  4679 (88%)  6479 (88%)  3876 (89%)  2428 (94%)  1050 (93%) | 0.78 (0.68, 0.90)  0.002  0.86 (0.75, 0.97)  0.004  0.70 (0.59, 0.82)  *  1.17 (1.03, 1.34)  *  2.30 (1.92, 2.74)  0.001  0.95 (0.80, 1.10)  ns | 1617 (75%)  2922  (71%)  4069  (72%)  2454 (73%)  1407 (72%)  639 (74%) | 0.95 (0.92, 0.98)  *  1.01 (0.91,1.12)  ns  0.97 (0.86, 1.10)  ns  1.06 (0.95, 1.18)  ns  1.16 (1.03, 1.32)  0.005  0.78 (0.65, 0.98)  * | 1527 (56%)  2991 (53%)  4268 (55%)  2509 (56%)  1575 (56%)  706 (57%) | 0.95 (0.92,0.99)  0.002  0.97 (0.90, 1.05)  ns  1.10 (1.05, 1.16)  *  0.96 (0.92, 0.99)  0.01  1.19 (1.08, 1.30)  0.003  0.88 (0.70,1.08)  ns | 1714 (66%)  3495 (67%)  4992 (69%)  2995 (70%)  1690 (67%)  768 (71%) | 0.90 (0.81, 0.99)  *  0.99 (0.91, 1.08)  ns  1.25 (1.17, 1.34)  *  1.19(1.11, 1.27)  *  0.99 (0.95, 1.02)  ns  0.95 (0.80,1.12)  ns | 2254  (85%)  4539 (86%)  6370 (87%)  3792 (88%)  2333 (90%)  1024 (91%) | 0.80 (0.70, 0.91)  0.04  0.86 (0.76, 0.97)  0.001  0.92 (0.80, 1.06)  ns  0.98 (0.97, 1.00)  ns  1.42 (1.22, 1.66)  ns  0.90 (0.80, 1.08)  ns |
| **Co-morbidities∞**  Cerebrovascular disease  Peripheral vascular disease  CKD  Chronic lung disease | 387 (84%)  125 (82%)  1116 (84%)  356 (81%) | 0.64 (0.49, 0.82)  0.005  0.56 (0.37,0.85)  *  0.60 (0.51, 0.70)  0.01  0.53 (0.42, 0.69)  * | 249 (69%)  104 (81%)  741 (74%)  229 (70%) | 0.89 (0.70,1.11)  ns  0.88 (0.81,0.96)  *  0.96 (0.92, 1.01)  ns  0.92 (0.72, 1.17)  ns | 249 (51%)  80 (51%)  502 (36%)  227 (49%) | 0.87 (0.72, 1.04)  ns  0.87 (0.63, 1.19)  ns  0.41 (0.37, 0.46)  *  0.79 (0.65, 0.95)  * | 306 (67%)  100 (67%)  887 (67%)  171 (40%) | 0.82 (0.75, 0.99)  *  0.99 (0.70, 1.40)  ns  0.99 (0.96, 1.04)  ns  0.30 (0.25, 0.37)  * | 401 (87%)  135  (89%)  1117 (84%)  360 (82%) | 0.80 (0.67, 0.95)  *  0.82 (0.70, 0.99)  *  0.73 (0.62, 0.85)  *  0.66 (0.51, 0.85)  0.001 |

**∞** Reference= those without the risk factor/co-morbidities

**p*<0.0001

ns not significant
